# Diagnosing Anaerobic Digesters' Function and Performance: From Meta‐Omics to Integrated Meta‐Omics Analyses

**DOI:** 10.1111/1758-2229.70417

**Published:** 2026-09-24

**Authors:** Kevin Iyere Ehiosun, Olivier Chapleur, Laurent Mazéas

**Affiliations:** ^1^ Université Paris‐Saclay, INRAE, PRocédés Biotechnologiques Au Service de L'environnement Antony France

**Keywords:** anaerobic digestion, biomarker, biomethane, meta‐omics, meta‐omics integration, multi‐meta‐omics

## Abstract

Anaerobic digestion (AD) of organic wastes by microorganisms into biomethane contributes significantly towards bioenergy generation. However, the bioprocess remains challenging due to its complex microbial ecology, dynamic biochemical pathways and sensitivity to perturbations. Over the past decade, using meta‐omics like metataxonomics, metagenomics, metatranscriptomics, metaproteomics and metabolomics to diagnose its functioning has become necessary and common. However, a single meta‐omics method can only provide limited biological understanding of the bioprocess. This led to the use of multi‐meta‐omics analyses in tandem; however, most studies still examine and interpret each meta‐omics separately, limiting their ability to uncover functional interactions across molecular levels. Currently, the frontier is integrated meta‐omics, where multi‐meta‐omics and process data are systematically combined into a single and interpretable system to unlock mechanistic understanding, diagnostic and predictive control of AD. This review critically examines specific application of meta‐omics in AD, discussing their strengths, limitations and distinct position in integrated meta‐omics approach. Importantly, it explores the computational frameworks, methodologies and challenges of integrated meta‐omics. Discovering diagnostic biomarkers with high predictive power and transferability across AD systems through cross‐omics validation is highlighted. As workflows standardise and technologies mature, integrated meta‐omics would significantly contribute to the advancement of AD bioprocess for bioenergy.

## Graphical abstract

The integration of multiple meta‐omics can tremendously improve our understanding of the structure, function and dynamics of the complex biological system in anaerobic digesters. Created in BioRender. https://BioRender.com/u9rj8ko.

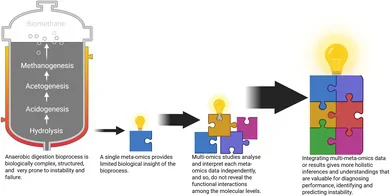

## 1 Introduction

A global development of bioenergy as an important part of renewable and sustainable energy (Scarlat and Dallemand 2019) is growing because of the finite fossil fuel reserves, the negative environmental impacts of using fossil fuels and the need for biomass and waste valorisation (Liu and Huang 2024; Igliński et al. 2024). Anaerobic digestion (AD) remains an important bioprocess for bioenergy generation and waste valorisation, while contributing to carbon neutrality and a circular economy (Capson‐Tojo et al. 2020). AD is the anaerobic degradation of organic wastes such as sewage, wastewater, agricultural residues, food waste, municipal solid waste and animal manure by a complex network of microorganisms to produce nutrient‐rich digestate and biogas that is upgraded into biomethane. Thus, in recent times, there has been extensive research and development in AD technologies and an increase in the global operation of full‐scale AD facilities.

However, AD is a highly structured and dynamic bioprocess, where intricate microbial, biochemical, syntrophic interactions and physiochemical processes intersect. This complexity makes the AD bioprocess a biological ‘blackbox’ (Roopnarain et al. 2021) that is very sensitive and susceptible to perturbations and eventual failure. Traditionally, physiochemical indicators such as pH, temperature, biogas/methane production, ammonia and volatile fatty acids (VFAs) concentrations, chemical oxygen demand (COD) etc. are used to track, control and optimise the functioning of an anaerobic digester (Kumar and Samadder 2020; Wu et al. 2021). While these physiochemical indicators are very important, they often are endpoints of microbial activities that indicate instability after it has already occurred (Krohn et al. 2022; Nie et al. 2023). Microbes are the backbone of the AD bioprocess and understanding their dynamics, functional interactions and the outcomes from such interactions is essential. But the microbiota that drives the AD bioprocess is very vast and complex. Furthermore, mechanistic explanation of microbiological events occurring inside the digester cannot be provided by physiochemical indicators alone. Hence, studying the AD's complex microbial ecology and the interplay with operational conditions has recently become an approach to understanding and improving the design, efficiency and stability of the AD bioprocess (Hashemi et al. 2021; Favale et al. 2025). This approach is rapidly advancing due to the enormous developments in the field of meta‐omics that have revolutionised our ability to understand the AD microbiomes and bioprocess at molecular resolution.

**FIGURE 1:**
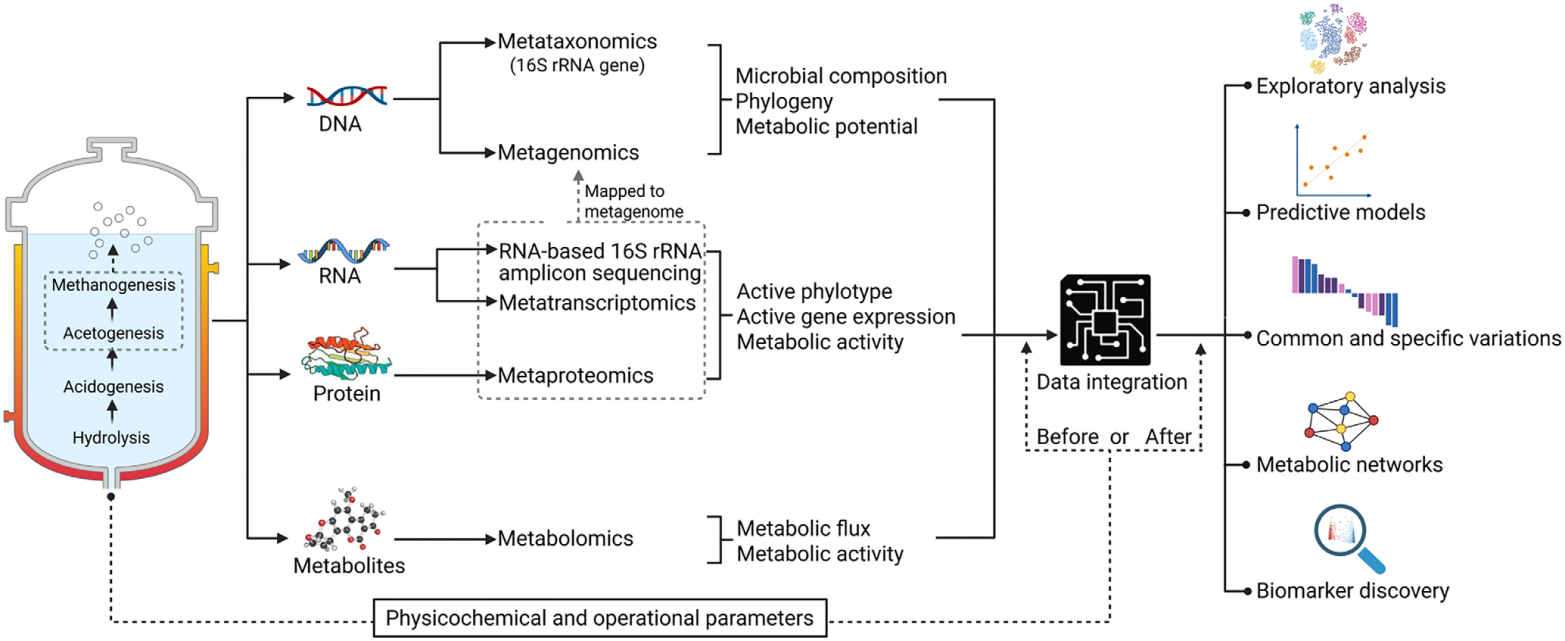
The combined use of metataxonomics, metagenomics, metatranscriptomics, metaproteomics and metabolomics alongside the digester's operational parameters for holistic comprehension of its complex microbiota structure, function and dynamics. This would aid in monitoring performance, predicting and identifying instability. Created in BioRender: https://BioRender.com/u9rj8ko.

Over the past decade, meta‐omics have enabled the characterisation of the AD bioprocess at the microbial composition, functional and activity levels as shown in Figure 1. While a single meta‐omics method can provide some limited biological understanding of the AD bioprocess, multi‐meta‐omics analyses enable studying the bioprocess across multiple molecular levels in tandem, providing a wider or deeper coverage. This helps in identifying which microorganisms are present, which genes are actively being expressed and their functional activities. Nevertheless, the field is transitioning towards integrated meta‐omics, where multiple meta‐omics layers are systematically combined into a single and interpretable system, alongside physiochemical and operational data, to reveal the causal chains that connect molecular events and outcomes to digester performance. This enhances mapping the interactions between the microorganisms and the bioprocess, design of metabolic networks, robust biomarker discovery and development of predictive models. While several studies on the AD bioprocess have applied multi‐meta‐omics, where each meta‐omics data is analysed independently, data integration is still very rare.

This review critically discusses meta‐omics and integrated meta‐omics application to AD, highlighting how multi‐meta‐omics datasets reveal microbe‐function networks, diagnose process instability and elucidate inhibition mechanisms. Furthermore, computational frameworks and approaches and key challenges for meta‐omics integration are examined.

## 2 Application of Meta‐Omics in AD Bioprocess

Meta‐omics enables studying the microbiome of the AD without the need for tedious and unrealistic culturing techniques. Recent research efforts are supported by the availability of high‐throughput sequencing technologies, high‐resolution mass spectrometers, established reference libraries or databases and faster computational and statistical methods (Long et al. 2021). Table 1 summarises the common meta‐omics in AD in terms of their specificity, strengths and limitations. Currently, a larger portion of published studies in AD bioprocess utilise a single meta‐omics approach to understand the complex underlying biological process. However, a single meta‐omics offers only partial snapshots of AD function. Metagenomics identifies genes but cannot confirm whether they are expressed. Metatranscriptomics reveals expression but not protein activity. Metaproteomics shows functional proteins but not metabolic flux. Metabolomics captures biochemical outcomes but not their microbial origins. Furthermore, most single meta‐omics and multi‐meta‐omics studies are still solely or heavily dependent on DNA analysis comprising either marker‐gene analysis (metataxonomics) or whole‐genome analysis (metagenomics). Reasons for this overly dependence on DNA‐centric techniques could be the relative availability, sensitivity and ease of this technology that makes it possible to analyse complex microbial communities almost in real time (Krohn et al. 2022; Tamames et al. 2024). Metataxonomics (16S rRNA amplicon sequencing) remains the principal method used to analyse bacterial and archaeal communities within the AD bioprocess despite its constraint to only taxonomic information with limited resolution (Kim et al. 2022). Exploring lower taxonomic levels such as genus and species is very important to better understanding the AD microbial community.

**TABLE 1:** Comparison of meta‑omics in anaerobic digestion.

Meta‐omics	What it measures	Strengths	Limitations
Metataxonomics	Amplicon‐based taxonomic profiling (16S, 18S, ITS). Community structure. Active phylotype (cDNA).	Cost‐effective, scalable and fast. High sensitivity for bacteria/archaea. Time‐series tracking.	Limited taxonomic resolution. No functional insight. Primer and amplification bias. Cannot distinguish live and dead cells, except if active phylotypes (cDNA).
Metagenomics	DNA Genetic potential. Taxonomic composition. Functional genes.	Comprehensive taxonomic profile. Potential functional profile. New microbes and genes identity.	Lack of functional activity. Assembly and binning challenges. Data complexity. High computational demand. Cannot distinguish live vs. dead.
Metatranscriptomics	RNA. Active gene expression. Functional guild activity.	Real time functional activity. Stress responses. Syntrophic coordination.	RNA instability High noise High temporal variability Data complexity. Mapping challenges
Metaproteomics	Functional Proteins (enzymes) Functional guild activity.	Direct measurement of functional enzymes. Links expression to catalytic function. Functional guild resolution. Expressed stress‐response proteins.	Extraction challenges. Sparse protein databases. Quantification difficulty.
Metabolomics	Metabolites Metabolic flux Biochemical outputs.	Metabolic flux Metabolic biomarkers	Extraction challenges. Matrix effects. Limited metabolite coverage. Ambiguous metabolite origin. Identification bottlenecks.

*Note:* Their strengths and limitations are complementary, making meta‑omics integration essential in revealing a systems‑level view of anaerobic digestion microbiome.

While meta‐omics provides high‐resolution insight into microbial structure, activity and metabolic outputs, these molecular signals only gain functional meaning when contextualised against digester conditions such as organic loading rate (OLR), hydraulic retention time (HRT), temperature, pH, alkalinity, ammonia, VFA profiles and biogas composition (Long et al. 2021). Linking meta‐omics layers with bioprocess data enables the identification of underlying relationships between microbial responses and performance outcomes; revealing how shifts in gene expression, enzyme production or metabolite accumulation correspond to inhibition events, pathway switching or changes in biomethane yield (Ruiz‐Sánchez et al. 2018). This integrated perspective is crucial for diagnosing instability, forecasting process upsets, optimising co‐digestion strategies and developing AI‐driven digital twins capable of real‐time control. Ultimately, coupling meta‐omics with operational and physiochemical datasets provides the systems‐level understanding required to engineer robust, adaptive and high‐performance AD systems.

### 2.1 Understanding AD Through Meta‐Omics

**FIGURE 2:**
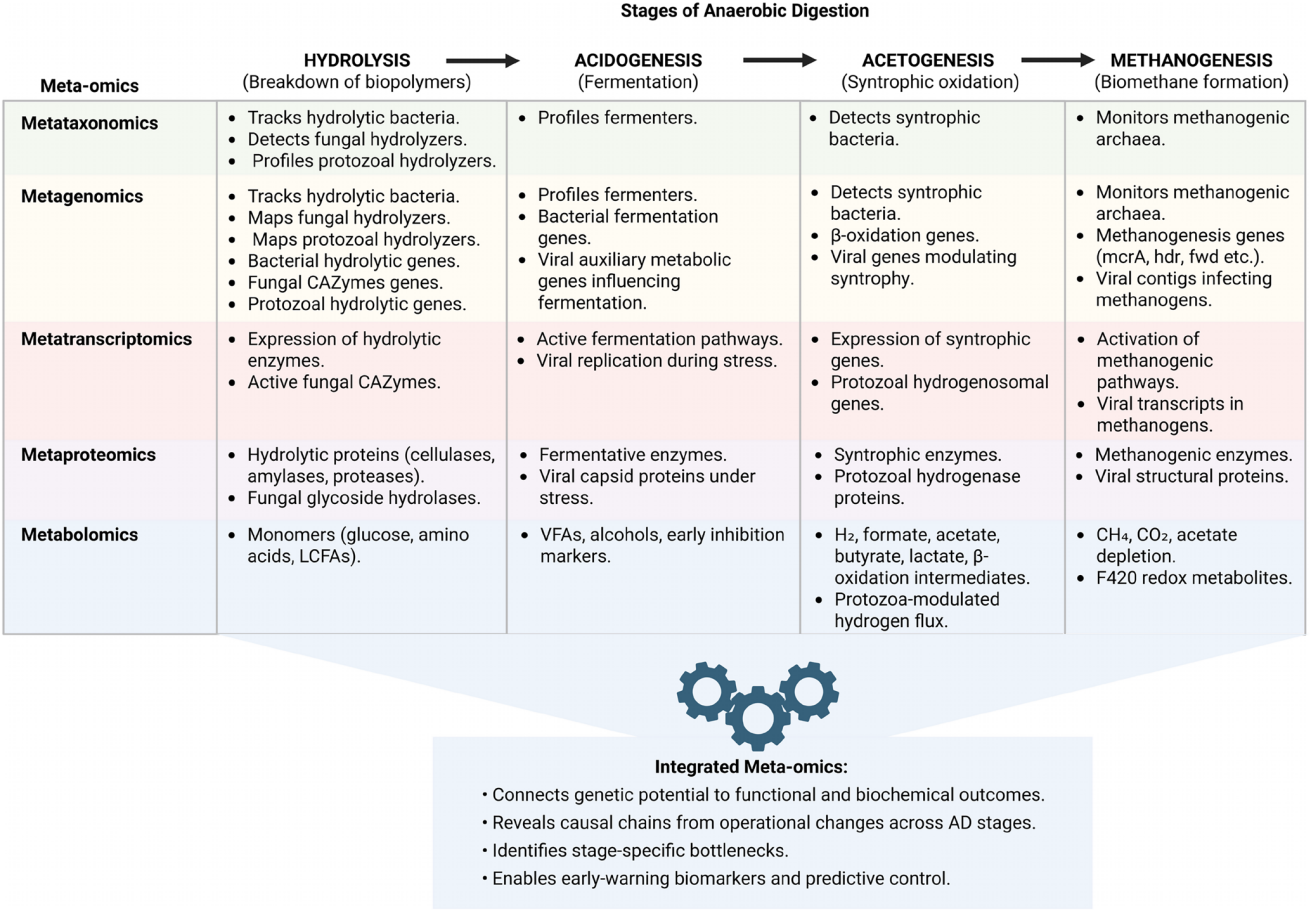
Meta‐omics mapping across the four stages of anaerobic digestion, including contributions from viruses, fungi and protozoa. Hydrolysis is driven by bacterial and fungal CAZymes; acidogenesis by fermentative bacteria, fungi and virus‐modulated pathways; acetogenesis by syntrophic bacteria and protozoal hydrogenosomes; and methanogenesis by archaea influenced by viral infection dynamics. Metataxonomics tracks community shifts, metagenomics reveals functional genes, metatranscriptomics captures real‐time activity, metaproteomics identifies active enzymes and metabolomics quantifies pathway outputs. Collectively, these layers provide a complete systems‐level view of AD microbiome structure, function and stability. Created in BioRender: https://BioRender.com/6egcwj8.

AD comprises four sequential steps with synergistic interactions, namely hydrolysis, acidogenesis, acetogenesis and methanogenesis occurring under anoxic conditions (Adekunle 2015; Meegoda et al. 2018). Each stage is intricately mediated by unique functional microbiota and tightly coupled biochemical pathways (Meegoda et al. 2018). A mechanistic understanding of AD requires examining how microbial functions unfold across its four stages and how these functions emerge from coordinated molecular interactions linking DNA, RNA, proteins and metabolites. Integrated meta‐omics provides the framework capable of resolving the molecular mechanisms that govern these transitions (Figure 2).

Fundamentally, metataxonomics and metagenomics have aided in characterising the meta‐genomic repertoire of hydrolytic bacteria, fermenters, syntrophic acetogens and methanogens; revealing the structure, diversity and functional potential encoded within the community (Vanwonterghem et al. 2014; Campanaro et al. 2020; Hu et al. 2024). These approaches have helped to define the core microbial community that drives the AD bioprocess (Calusinska et al. 2018; Bedoya et al. 2020; Jiang et al. 2021; Dueholm et al. 2024) and in the development of the Microbial Database for Activated Sludge and Anaerobic Digesters (MiDAS) (Dueholm et al. 2024). The core microbiota refers to a set of microbial taxa that are consistently present across functioning anaerobic digesters (Fujimoto et al. 2019), despite microbial differences that are principally caused by substrate types, inoculum source and operational conditions (De Vrieze, Raport, et al. 2016). This core microbial community makes the AD a balanced ecological environment for a structured metabolic process (Krohn et al. 2022). In fact, studies have shown that microbial entry from feeding substrates can partially change the microbial composition of AD (Wang, Yu, et al. 2018) but might not significantly contribute to the functionality of the AD (Jiang et al. 2021). Exploring this microbiota‐functionality nexus reveals core microbiota that correlate with biogas production (Tao et al. 2020). The most studied AD microbiome is the hydrolytic and fermentative bacteria and methanogenic archaea (Theuerl et al. 2019). Hydrolytic bacteria secrete extracellular enzymes during hydrolysis to convert organic polymers such as carbohydrates, proteins and lipids into soluble monomers like sugars, amino acids and long chain fatty acids (LCFAs), respectively. While most of the reported hydrolytic bacteria belong to the *Firmicutes* and *Bacteroides* phyla (Lim et al. 2020), the genera *Clostridium, Bacteroides, Symbiobacterium*, *Bacillus, Lactobacillus* etc. are commonly reported as the core bacteria involved in hydrolytic degradation and fermentation of cellulose, polysaccharides and proteins (Wang, Wang, et al. 2018; Lam et al. 2021). Acidogens generate intermediary products like VFAs, hydrogen, carbon dioxide, ammonia during the fermentation of products from the preceding hydrolytic stage. Thereafter, acetogens convert the organic acids into acetic acid, carbon dioxide and hydrogen for methanogenesis. During methanogenesis, methanogens, which are archaea, can produce methane from acetic acid, from carbon dioxide and hydrogen or from methyl compounds. Hence, methanogens are categorised into (i) acetotrophic or acetoclastic (ii) hydrogenotrophic and (iii) methylotrophic methanogens (Lim et al. 2020). Remarkably, any of the methanogenic pathways can be dominant depending on the state of the digester (Cardona et al. 2019).

However, metagenomic abundance does not directly imply gene expression or microbial activity (Jia et al. 2018; De Vrieze, Regueiro, et al. 2016). For instance, acetogenesis, the most thermodynamically constrained stage, depends on syntrophic bacteria whose activity cannot be inferred from community composition alone (Heyer et al. 2019). Furthermore, some methanogens such as members of the order *Methanosarcinales* are versatile and known to have genes for multiple methanogenic pathways. Consequently, the use of only DNA‐centric analysis (metataxonomics and metagenomics) struggles in distinguishing which pathway is actively expressed (Ni et al. 2022). A comprehensive view of microbial activity is achieved by linking microbial presence to their functional roles via functional analysis under specific operational and physiochemical conditions (Van Den Bossche et al. 2025). Metatranscriptomics helps in identifying active microbial clusters and functions in the digester by capturing real‐time gene expression patterns. This uncovers how the microbial guilds dynamically regulate hydrolysis, fermentation, β‐oxidation, syntrophic acetate oxidation (SAO) and methanogenesis in response to operational and environmental pressures. For example, during hydrolysis, transcripts encoding cellulases, proteases and lipases increase in response to complex substrates, while acidogenic conditions trigger upregulation of fermentation genes such as lactate dehydrogenase or pyruvate‐formate lyase (Jia et al. 2018; Herold et al. 2020). Metaproteomics adds another functional layer by identifying the functional proteins and enzymes actually deployed in situ, thereby linking transcriptional signals to catalytic capacity and pathway execution (Hassa et al. 2018). This is particularly important in AD, where post‐transcriptional regulation is common and mRNA levels do not always correlate with protein abundance (Samih et al. 2026). Metaproteomic data reveals the functional machinery responsible for β‐oxidation, SAO, hydrogenase activity and methanogenesis. To mention a few, the detection of methyl‐coenzyme M reductase subunit A (mcrA) confirms active methane formation, while the presence of formate dehydrogenases and [FeFe]‐hydrogenases indicates active syntrophic electron transfer (Zeng et al. 2024).

It is metabolomics that captures the biochemical outcomes of the molecular processes by detecting and quantifying LCFAs, lipids, sterols, phenolics, amino acids, polysaccharides, purines, nucleotides and complex microbial metabolites, along with their β‐oxidation intermediates, TCA‐derived fermentation intermediates, VFAs, redox cofactors and stress‐response metabolites (Yang et al. 2014). These metabolites represent the emergent phenotype of the system and provide real‐time indicators of pathway fluxes, bottlenecks and inhibition. However, matrix effects and metabolite identification bottlenecks are major challenges for metabolomics (de Jonge et al. 2022).

### 2.2 Emerging Roles of Non‐Bacterial and Non‐Archaeal Microbiota

AD research has historically focused on bacteria and archaea; however, viruses, fungi and protozoa represent a substantial but understudied fraction of AD microbiota. Recent advances in meta‐omics now make it possible to illuminate their ecological functions, metabolic contributions and interactions with classical AD guilds (Figure 2).

Viral communities, particularly bacteriophages and archaeal viruses (Ngo et al. 2022), shape microbial turnover through ‘kill‐the‐winner’ dynamics, modulate syntrophic and methanogenic populations and facilitate horizontal gene transfer (Dar et al. 2025; Guo et al. 2025; Shi et al. 2024). Metataxonomics, metagenomics and metatranscriptomics increasingly detect the presence of lytic and lysogenic viruses (mainly dominated by *Siphoviridae* and *Podoviridae*), as well as viral replication during ammonia or LCFA stress (Fan et al. 2023; Bhattarai et al. 2024). Metaproteomics and metabolomics can capture the lysis‐driven shifts in intracellular metabolite pools. Anaerobic fungi, especially members of the *Neocallimastigomycota*, contribute potent carbohydrate‐active enzymes that enhance the hydrolysis of lignocellulosic substrates (Gruninger et al. 2014; Dollhofer et al. 2018). Meta‐omics approaches have begun to uncover fungal CAZyme gene clusters, active cellulase and xylanase expression and glycoside hydrolase proteins that complement bacterial hydrolytic pathways (Swift et al. 2019). Similarly, protozoa (mainly ciliates, flagellate and amoeba) play roles in predation, hydrogen metabolism and symbiosis with methanogens (Prabhakaran et al. 2016). Meanwhile, metagenomics and metatranscriptomics detect hydrogenosomal genes and active protozoal metabolism in similar anaerobic systems like the rumen, while metabolomics reveals protozoa‐driven shifts in hydrogen and VFA profiles (Hirakata et al. 2019; Lu et al. 2025; de Almeida et al. 2018).

Together, these emerging insights highlight the need to incorporate viruses, fungi and protozoa into systems‐level models of AD ecology. Meta‐omics provides the resolution required to characterise their genomes, transcripts, proteins and metabolites in parallel with bacteria and archaea, while meta‐omics integration frameworks enable analysing their data together. As these non‐classical taxa become better understood, they are likely to reshape our understanding of hydrolysis efficiency, syntrophic resilience, inhibition dynamics and biomethane yield. This would ultimately improve the predictive and mechanistic power of AD microbiome research.

### 2.3 Tracking Inhibitions and Instability Through Meta‐Omics

AD bioprocess is highly susceptible to disturbances or inhibitors such as ammonia, hydrogen sulphide and heavy metals, most of which are substrate induced (Mutegoa and Sahini 2023; Shakeri Yekta et al. 2019; Puig‐Castellví, Cardona, Bureau, et al. 2020). While changes in temperature could arise from operational parameters or environmental influences (Theuerl et al. 2019), pH is directly and indirectly substrate induced. These important factors shape the structure and function of the AD microbiota. In recent times, discovering the molecular indicator of specific instabilities is becoming essential and predicting AD failure using these bioindicators is of utmost importance (Lee et al. 2017).

#### 2.3.1 Ammonia Inhibition

Ammonia inhibition remains one of the most pervasive and operationally significant challenges in AD bioprocess, affecting both community structure and functional performance (Jiang et al. 2021; De Vrieze et al. 2015). Ammonia is necessary for microbial growth, but it can inhibit AD if present at high concentration. Total Ammonia Nitrogen (TAN) in AD comprises of Free Ammonia Nitrogen (FAN), NH_3_ (unionised form) and NH_4_^+^ (ionised form), with FAN regarded as the major inhibitor and most toxic (Liu et al. 2019). Although there is no consensus in the literature on the inhibitory limits of ammonia, amounts over 1000–1500 mg TAN‐N/L are reportedly inhibitory (Capson‐Tojo et al. 2020). Food waste, livestock products and manure can have an enormous amount of nitrogenous materials such as proteins, urea or nucleic acids (Buhlmann et al. 2019), which can elevate the concentration of ammonia after degradation (Lam et al. 2021). The response of the AD microbiota to ammonia depends mainly on pH and temperature, as well as the microbial composition (Capson‐Tojo et al. 2020; Jiang et al. 2021). Ammonia inhibition principally affects methanogenesis (Buhlmann et al. 2019; Poirier et al. 2016). Metataxonomics and metagenomics provide the baseline in identifying the dynamics of the methanogenic guilds that are most sensitive to ammonia, particularly acetoclastic taxa such as *Methanosaeta*. Additionally, they reveal the presence of syntrophic acetate‐oxidising bacteria (SAOB) that often become dominant under high‐ammonia conditions (Puig‐Castellví, Cardona, Bureau, et al. 2020; Ruiz‐Sánchez et al. 2018). Shifts towards fermentative dominance and reduced syntrophic taxa are early structural indicators of imbalance (Ostos et al. 2024). Metatranscriptomics presents ammonia inhibition by detecting downregulation of acetoclastic methanogenesis genes (e.g., *mtaA*, *acs*, *cdh*) and upregulation of hydrogenotrophic pathways, stress‐response genes and ammonium transporters, often before biomethane yield declines (Gaspari et al. 2024; Fischer, Güllert, et al. 2019; Fischer, Ulbricht, et al. 2019). These metatranscriptional shifts reflect the community's attempt to reconfigure electron flow away from acetate cleavage towards syntrophic acetate oxidation coupled to hydrogenotrophic methanogenesis (Ni et al. 2022; Wang et al. 2022). The hydrogenotrophic pathway is considered more resilient to the acetoclastic pathway during ammonia stress (Poirier et al. 2016; Ruiz‐Sánchez et al. 2019). While the shift to hydrogenotrophic methanogenesis sustains AD's stability and resilience by maintaining a low hydrogen partial pressure, it can still succumb to ammonia inhibition (Peng et al. 2018). Metaproteomic profiles typically display reduced abundance of key acetoclastic enzymes such as acetyl‐CoA decarbonylase/synthase and mcrA, alongside increased expression of hydrogenases, formate dehydrogenases and SAO‐associated β‐oxidation enzymes (Lam et al. 2021; Zhang et al. 2022). These protein‐level signatures provide the direct evidence of functional reorganisation and can be linked to thermodynamic constraints imposed by elevated ammonia concentrations.

Ultimately, metabolomics shows the consequences of these molecular changes, comprising accumulation of key metabolites such as pyruvate, acetate, xanthine, alanine, succinate, branched VFAs (isobutyrate, isovalerate), lactate, cofactors, etc. (Chapleur et al. 2021; Wang, Campuzano, et al. 2024; Murovec et al. 2018; Jiao et al. 2026). These metabolites are central intermediates in glucose metabolism, amino‐acid metabolism, purine degradation and the TCA‐derived fermentation pathways. Their accumulation indicates electron flow blockage, redirection of carbon flux towards reduced end‐products, impaired syntrophic oxidation and methanogenesis inhibition, forcing microbes into stress‐response modes (Amha et al. 2018). Particularly, VFAs oxidation is synergistically impacted by high levels of ammonia, which leads to its' accumulation (Chapleur et al. 2021), acidification of the system and inhibition of syntrophs and methanogens.

#### 2.3.2 Sulphide Inhibition

Sulphide (H_2_S/HS^−^) inhibition, arising primarily from the reduction of sulphate and the degradation of sulphur‐containing amino acids, represents another major biochemical and operational challenge. The levels of sulphide and saturated long‐chain fatty acid in a digester selectively drive the formation of LCFA‐degrading microbiota (Shakeri Yekta et al. 2019). Hydrogen sulphide toxicity is pH‐dependent, with the more toxic form (H_2_S) at pH < 6 (Paulo et al. 2015). Elevated sulphide concentrations exert toxicity on both bacteria and archaea by disrupting enzyme function, damaging metal‐containing cofactors, inhibiting electron transport and precipitating essential trace metals such as Fe, Ni and co‐factors required for methanogenic enzymes (Shakeri Yekta et al. 2019, 2014; Schnürer 2016). Actually, sulphate‐reducing bacteria (SRB) such as *Desulfovibrio*, *Desulfotomaculum* and *Desulfobulbus* compete with methanogens for both substrates and electron donors (H_2_, acetate, propionate) (Beale et al. 2016). Metagenomic profiling reveals the presence of methanogens sensitive to sulphide toxicity, particularly hydrogenotrophic taxa whose Ni‐Fe hydrogenases are highly susceptible to sulphide binding (Nomura et al. 2025). Metaproteomic profiles typically show reduced abundance of Ni‐Fe hydrogenases, formylmethanofuran dehydrogenase and coenzyme‐M reductase complexes essential for hydrogenotrophic and acetoclastic methanogenesis (Thanh et al. 2016). In parallel, SRB‐associated proteins involved in sulphate activation and reduction (e.g., APS reductase, DsrC) increase in abundance, confirming the functional dominance of sulphate reduction pathways. These provide direct evidence of sulphide‐driven shifts in electron flow and metabolic competition. The precipitation of metal‐sulphide complexes (e.g., FeS, NiS) is detectable by metabolomics through characteristic shifts in VFAs, redox cofactors, H_2_ accumulation and impaired acetate conversion.

#### 2.3.3 LCFAs Inhibition

LCFAs (oleate, palmitate, stearate etc.) are important metabolites produced from lipids hydrolysis that require specialised β‐oxidising bacteria for degradation. Their degradation is very essential towards achieving functional stability of the AD. LCFA degradation is carried out by obligate LCFA‐oxidising bacteria such as *Syntrophomonas*, *Syntrophus*, *Syntrophothermus*, etc. into acetate, hydrogen and CO_2_ for methanogenesis (Nakasaki et al. 2020). However, LCFAs accumulation can result from either their increased production or reduced degradation rates, which signals a dysfunction. Due to the hydrophobic property of LCFA and the slow growth rate of LCFA‐degraders, degradation proceeds slowly. In addition, LCFAs can accumulate owing to a feedback inhibition on syntrophic VFA‐oxidising bacteria (Wu et al. 2021). In particular, LCFAs accumulation inhibits syntrophs and methanogens (Amha et al. 2018). Thus, LCFA overload can be traced through downregulated β‐oxidation transcripts, upregulated stress‐response genes linked to membrane damage, reduced hydrogenase activity and VFAs accumulation. LCFAs accumulation causes sludge foaming, disruption of microbial community structures and then operational instability (Shakeri Yekta et al. 2019).

#### 2.3.4 Seasonal and Temperature Fluctuations

Other forms of disturbances could occur from seasonal changes, temperature fluctuations or changes in operational parameters such as HRT, OLR, etc. Seasonal changes can have a significant effect on feedstock characteristics, temperature and ultimately biomethane production (Sposob et al. 2020). The operating temperature is an important factor in shaping the microbiota, functional pathways, biomethane yield and bioprocess stability (Hupfauf et al. 2018). Although digesters are generally operated at 35°C (mesophilic) and 55°C (thermophilic), some digesters are reported to operate at ambient temperature (Meegoda et al. 2018). The latter are more susceptible to seasonal temperature fluctuations and biomethane production. In any case, meta‐omics studies have elucidated the microbial community structure and function during temperature fluctuations (Sposob et al. 2020; Bahadur et al. 2024). In one study, some thermotolerant consortia were identified to drive quick recovery during abrupt temperature change from 35°C to 55°C and vice versa (Madigou et al. 2019). Furthermore, temperature change (mesophilic to thermophilic) highlighted a correlation between methanogens and bacteria that maintained the AD stability (Lv et al. 2019). The stability and flexibility of microbial community in AD is vital to coping with the stress from temperature changes. However, stability and flexibility are better understood by incorporating functional analysis (metaproteomics and metatranscriptomics) to taxonomic profiling. This reveals unique functional redundancy among the AD microbiota (Gunnigle et al. 2015), where different microbial groups perform the same functions under different temperatures. Stability during temperature fluctuation can occur through relatively upregulating methanogenesis, differential expression of methanogenic proteins and diminishing other functional pathways (Gunnigle et al. 2015). As such, biomethane production can remain relatively stable despite temperature change and microbial fluctuations (Bedoya et al. 2020). Indeed, a connection exists between the microbial diversity in AD and the bioprocess robustness.

### 2.4 Evaluating AD Bioprocess Performance During Optimization Through Meta‐Omics

Over the years, efforts have been made in improving the efficiency and resilience of the AD bioprocess. Such improvements include redesign of biodigesters, pre‐digestion treatments, co‐digestion, micronutrient supplementation, addition of carbon‐based materials, etc. How these improvements affect the overall efficiency and robustness of the AD bioprocess is better explained by meta‐omics.

While traditional digesters still exist, modified versions and new designs are increasing. Modifications are aimed at improving mixing regimes, staged digestion, enhanced retention of slow‐growing methanogens, etc. Meta‐omics provides a mechanistic foundation for redesigning digesters by revealing how structural and operational changes influence microbial guilds and metabolic fluxes. An example is the development of ‘Temperature Staging and Biological Phasing’ (TSBP) bio‐digester that enables controlling the temperatures of the hydrolysis and methanogenesis phases separately (Zhao et al. 2022). Another example is the design of an anaerobic labyrinth‐flow bioreactor (AL‐FB), which partitions acidogenesis and methanogenesis while retaining microbial biomass, prolonging HRT and diminishing sludge flotation (Zieliński et al. 2023). In both cases, metagenomics and functional enzymes annotation identified functional changes in hydrolytic, fermentative, syntrophic and methanogenic pathways. This enables targeted redesigns that ensure structural modifications translate into functional improvements.

The hydrolysis phase is regarded as the rate limiting step in AD bioprocess. High cellulose and/or lignin‐containing biomass products could be difficult to hydrolyse by hydrolytic bacteria (Chettri et al. 2023). Furthermore, wastes containing high levels of suspended solids like fat, oil and grease (FOG) can inhibit microbial development (Usman et al. 2020). Hence, a pre‐treatment step is often applied to optimise hydrolysis (Zhao et al. 2019; Chen, Ping, et al. 2022). Hydrothermal, physical, chemical, thermal or biological pre‐treatment can effectively breakdown waste, improve their bioavailability and reduce ammonia level (Li et al. 2018). Metagenomics and metataxonomics have shown that degradation of thermally hydrolysed sludge caused significant reduction in microbial diversity and shaped the core functional guilds with strong interspecies interactions (Zhang et al. 2021). Likewise, they revealed that the activity of *Methanosaeta* increased 5‐folds after biological pre‐treatment, significantly enhancing biomethane production (Zhao et al. 2019). While metaproteomics detects increase in cellulases, xylanases and β‐oxidation enzymes following effective pre‐treatment, metabolomics tracks the reductions in recalcitrant intermediates (Chirania et al. 2022).

Co‐digestion strategies also benefit from meta‐omics by enabling rational selection of feedstock combinations that complement microbial functional potential, bioprocess stability and biomethane yield. Co‐digestion is an approach that permits the simultaneous digestion of different types of organic waste. The systemic co‐digestion of cattle manure and unsaturated LCFA was reported (Gaspari et al. 2021). The relative abundance of hydrogenotrophic methanogens increased upon lipid supplementation and stability was maintained by syntrophic metabolism of fatty acids. Combining different substrates can complement nutrient deficiency that could limit biomethane production. Wastewater, for instance, has a low carbon to nitrogen ratio that limits digestion efficiency and biomethane yield (Cardona et al. 2019). While co‐digestion of wastewater with either grass or fish waste showed comparable changes in the community structure through metataxonomics (Puig‐Castellví, Cardona, Bouveresse, et al. 2020), metabolomics revealed that only co‐digestion with fish waste improved metabolic biodegradability of the wastewater (Puig‐Castellví, Cardona, Jouan‐Rimbaud Bouveresse, et al. 2020).

Changes in digester's performance due to micronutrients limitation are easily observable at the methanogenesis phase (Wintsche et al. 2018). Hence, supplementation of micronutrients such as manganese (Mn), nickel (Ni), tungsten (W), cobalt (Co) and molybdenum (Mo) involved in the function of essential enzymes in the methanogenic pathways have offered improved AD performance and stability (Evranos and Demirel 2015; González‐Suárez et al. 2018). The addition of elemental sulphur was reported to stimulate synergetic growth of sulphur disproportionation bacteria, acidogens and methanogens and an increase of vital functional enzymes (Qiao et al. 2025). Both metataxonomics and metaproteomics confirmed a shift from acetoclastic to hydrogenotrophic methanogenesis during trace elements (Co, Ni, Mo, Mn and W) starvation (Wintsche et al. 2018). Addition of carbon‐based materials such as activated carbon, zeolite, biochar and hydrochar has also become common because of their ability to particularly suppress ammonia stress via adsorption, enhance selective microbial colonisation and promote direct interspecies electron transfer. Zeolite contains Na^+^, K^+^, Ca^2+^ and Mg^2+^ needed for microbial growth, thus making it a good supplement. Zeolite reportedly promoted the development of propionate degraders, stimulated bacteria‐archaea syntrophy (Cardona et al. 2021), mitigated the effect of sodium chloride and erythromycin by promoting hydrolytic, fermentative bacteria and methanogens (Wang, Dürr, et al. 2024). Likewise, biochar enabled the selective colonisation of functional microbes like *Methanosarcina* and syntrophic bacteria while enhancing the production and degradation of intermediate acids (Luo et al. 2015) during ammonia stress (Lü et al. 2016). Although metagenomic analysis revealed that addition of hydrochar under ammonia inhibition mainly improved the diversity of hydrogenotrophic methanotrophs, metabolomics showed a shift from hydrogenotrophic methanogenesis to other pathways, such as methylotrophic and methanolic methanogenesis, which ultimately improved methane production (Wang, He, et al. 2024).

## 3 An Integrated Meta‐Omics Strategy for AD Bioprocess

Integrated meta‐omics considers each meta‐omics dataset as a data block. A data block is a two‐way matrix depicting samples and variables by rows and columns. Integration creates a multiblock, where each block is defined by its respective components. The inter‐relationships between the blocks can be modelled by establishing connections between their components. However, the complexity and heterogeneity of multi‐omics datasets present both technical and computational challenges that hamper biological interpretation and cross‐validation of results/models (Picard et al. 2021). Thus, a robust multi‐meta‐omics integration approach should address these profound challenges without compromising on reproducibility and scalability.

### 3.1 Developing Integrated Meta‐Omics Approach

Developing an integrated meta‐omics approach requires meticulous research design and planning, selection of appropriate meta‐omics methodology, data and meta‐data collections, statistical and computational considerations. Furthermore, the specific goals, research questions and hypotheses should be clearly defined as well as the scope and limitations of the study. Selecting the appropriate type and number of meta‐omics platforms to use should be based on the type and value of information desired, the domain knowledge, feasibility and costs (Arıkan and Muth 2023).

#### 3.1.1 Sample Collection

Ensuring the collection of DNA, RNA, proteins and metabolites simultaneously from the same sample is essential to facilitate direct cross‐omics comparisons. Similarly, the quality and consistency of sampling are vital towards generating reliable data across the different meta‐omics platforms. It is very important to follow standard protocols for sample collection, storage and preparation to lessen technical variability and batch effects. Another crucial step is deciding on the interval, frequency and duration of sampling. Collecting samples across extended durations often gives a more thorough analysis, especially for longitudinal or time‐series studies. It is imperative to decide on the need for controls and replicates including biological and technical controls/replicates. Each step is critical to ensuring the accuracy, reproducibility and biological interpretation and relevance of the study (Pinu et al. 2019).

#### 3.1.2 Data Pre‐Processing

Since meta‐omics generate heterogeneous datasets with different data formats, data size, data resolution, data units and scales, time scale, number of samples, noise levels and number of dimensions, each meta‐omics dataset should be pre‐processed before integration to improve comparison, reliability, reproducibility and interpretability. There are no benchmark workflows for pre‐processing meta‐omics data. Notably, each meta‐omics dataset is inherently different and unique, so pre‐processing methods or steps for each could be different (Chen, Li, and Xu 2022). Nonetheless, pre‐processing or data cleaning, includes but is not limited to subtraction of blanks, removal or imputation of missing values, correction for batch effects and removal of outliers. There are various statistical methods to manage missing data and batch effects. While some methods are quite simple, others are more robust but limitations and drawbacks still exist (Flores et al. 2023; Yu et al. 2024). Thus, selecting an appropriate method and evaluating the results is apparently challenging. Other pre‐processing steps involve data normalisation and transformation, data centering and scaling, data reformatting etc. The pre‐processing steps for microbiome data have been recently reviewed elsewhere (Zhou et al. 2023).

#### 3.1.3 Dimensionality Reduction

Dimensionality reduction is applied to relatively reduce the dimension of the feature space, that is, decreasing the number of features using the existing feature parameters (Meng et al. 2016). This reduces data complexity and redundancy and removes irrelevant or noisy information. This in turn reduces computational complexity and it can improve interpretability of models. Feature extraction and feature selection are two approaches of dimension reduction. Feature extraction transforms and/or combines the original features into new synthetic features, often with lower dimensions that capture essential structure, variance or correlations in the data. This helps in removing noise and redundancy while preserving only relevant information. By generating new features, feature extraction reveals latent structures and patterns that are helpful in uncovering complex relationships. However, biological interpretability or relevance could be lost or compromised after feature extraction because the newly generated features are typically mathematical abstractions (Jia et al. 2022). Feature extraction is achieved by using exploratory models such as Principal Component Analysis (PCA), Supervised Principal Component Analysis (SPCA), Correspondence Analysis (CA), Principal Coordinate Analysis (PCoA), cluster analysis, etc. or test like Partial Least Square (PLS), Fold change, etc. While feature extraction reduces dimensionality by transformation and/or combination, feature selection reduces dimensionality by retention. Feature selection methodically classifies and retains the most non‐redundant and significant features, while avoiding overfitting, for use in model construction. As such, interpretability is maintained as a sub‐set of the original data is preserved and overfitting is significantly reduced. Besides, feature selection can be dependent on prior knowledge or a hypothesis. However, feature selection may not capture all interactions and it requires validation. Feature selection is suitable for AD studies, where there are relatively more features and few samples (Jia et al. 2022). Filter‐based, embedded and wrapper‐based are the three classes of feature selection (Picard et al. 2021). Filter‐based methods evaluate features using statistical metrics such as correlation coefficients, variance and scores that are independent of the final model. Depending on established evaluation criteria, a threshold is used to retain and filter out features. Wrapper‐based methods use a predictive model to evaluate the importance of subsets of features that optimise the final model performance. While filter‐based methods are simple and common, the wrapper‐based methods are better but time and computationally demanding. Meanwhile, hybrid methods that combine filter‐based and wrapper‐based methods in order to balance accuracy and computational efficiency are becoming common (Theng and Bhoyar 2024). Embedded methods integrate feature selection into the model training process. Least absolute shrinkage and selection operator (Lasso), tree‐based models (Random Forest, Extreme Gradient Boosting (XGBOOST), etc.) and Gradient Boosting are well‐known embedded techniques. The R‐based package, mixOmics, is a multi‐block toolbox suitable for dimensional reduction of single and multi‐omics datasets by feature selection (Rohart et al. 2017).

### 3.2 Current Methodologies for Data Integration

#### 3.2.1 Integration Frameworks

Table 2 highlights the diversity of multi‐omics integration frameworks suitable for AD, ranging from linear latent‐space models to probabilistic frameworks, network‐based approaches, tensor‐based methods and deep learning architectures. Linear methods like PLS, Two‐way Orthogonal Partial Least Squares (O2PLS), Canonical Correlation Analysis (CCA), Common Dimensions (ComDim) or Common Components and Specific Weights Analysis (CCSWA), Nonnegative Matrix Factorization (NMF) and iClusterPlus assume straight‐line relationships across meta‐omics layers. They are easy to interpret but cannot capture the non‐linear, threshold‐driven and feedback‐regulated dynamics that occur in AD. They also require careful scaling because variable magnitudes directly affect the model. For instance, if metabolomics intensities are huge and metagenomics counts are small, linear models will overweight the large‐scale metabolomics, underweight subtle metagenomic signals, distort component loadings and misidentify shared variation. This is why deep learning methods like Variational Autoencoders (VAEs), Multiple Kernel Learning (MKL) and Deep Canonical Correlation Analysis (DCCA) are often better for non‐linear AD dynamics, but they require large datasets and careful regularisation (Picard et al. 2021). Meanwhile, ComDim or CCSWA offer highly interpretable multi‐block decompositions with explicit block contributions, making it ideal for AD (Cariou et al. 2019; El Ghaziri et al. 2016). DIABLO is a supervised multi‐block method based on sparse PLS and therefore relies on linear latent‐variable modelling (Singh et al. 2019). While it cannot capture non‐linear biological dynamics, it maximises cross‐omics covariance, which is biologically meaningful. Hence, it excels at identifying cross‐omics biomarker signatures linked to phenotypes, offering high interpretability and strong predictive performance. TimeOmics and TensorOmics uniquely address longitudinal and multi‐factor experimental designs that enable dynamic multi‐meta‐omics analysis critical for bioprocess monitoring (Bodein et al. 2022; Kodikara et al. 2026).

**TABLE 2:** Comprehensive multi‑omics integration frameworks suitable for anaerobic digestion.

Framework	Integration type	Core model/concept	Strengths	Limitations	References
DIABLO	Supervised multi‐block	Sparse PLS linking meta‐omics to phenotype.	Strong biomarker discovery. Interpretable. Handles many omics blocks.	Requires phenotype labels. Linear latent‐variable modelling.	Singh et al. (2019)
MOFA+	Unsupervised	Bayesian factor analysis.	Handles missing data Finds shared/specific factors.	Factor interpretation can be difficult.	Argelaguet et al. (2018, 2020)
iClusterPlus	Unsupervised joint latent space	Penalised latent variable model.	Integrated clustering with feature selection.	Captures only linear relationships. Tuning penalties is complex.	Mo et al. (2018)
JIVE/AJIVE	Unsupervised decomposition	Joint and individual variation.	Clear separation of shared and unique features.	Not predictive. Captures only linear relationships.	Feng et al. (2018)
TimeOmics	Longitudinal multi‐omics	Multi‐block PLS with clustering.	Captures temporal trajectories. Ideal for time‐series.	Requires dense time points.	Bodein et al. (2022)
TensorOmics	Tensor‐based longitudinal multi‐omics	Multi‐way decomposition.	Captures meta‐omics across time and conditions.	Computationally heavy. Need large and well‐aligned datasets. Assume linear or low‐rank relationships.	Kodikara et al. (2026)
ComDim or CCSWA	Multi‐block	Common latent dimensions and block weights.	Clear block saliences. Quantifies block contributions. Interpretable.	Sensitive to scaling. Captures only linear relationships. Sample size across blocks.	Cariou et al. (2019) and El Ghaziri et al. (2016)
Multi‐modal VAE	Deep learning	Variational autoencoders.	Non‐linear. Generative Handles high‐dimensional data.	Requires large datasets. Less interpretable.	Senellart and Allassonnière (2025)
CCA	Linear correlation	Maximises correlation.	Simple baseline. Interpretable.	Captures only linear relationships.	Wróbel et al. (2024) and Jendoubi and Strimmer (2019)
PLS/O2PLS	Latent space	Shared and orthogonal variation.	Good for pathway‐level integration.	Captures only linear relationships. Limited scalability.	Lê Cao et al. (2008) and el Bouhaddani et al. (2016)
NMF	Unsupervised	Non‐negative factorization.	Parts‐based and interpretable modules.	Requires non‐negative data. Captures only linear relationships.	Lin and Boutros (2020)
Graph‐based Integration	Network‐based	Multi‐layer biological networks.	Great for pathway inference.	Hard to scale. Sensitive to noise.	Valous et al. (2024)
MKL	Kernel‐based	Multiple kernel learning.	Non‐linear; strong prediction.	Requires careful kernel design.	Briscik et al. (2024)
WGCNA	Network modules	Co‐expression networks.	Module discovery. Phenotype association.	Not designed for multi‐omics by default.	Langfelder and Horvath (2008)
DCCA	Deep learning	Non‐linear CCA	Captures complex relationships.	Requires large datasets	Zuo et al. (2021)

*Note:* These frameworks form a comprehensive methodological system for integrating meta‐omics in AD and other complex systems.

Abbreviations: AJIVE: Angle‐Based Joint and Individual Variation; CCA: Canonical Correlation Analysis; CCSWA: Common Components and Specific Weights Analysis; ComDim: Common Dimensions; DCCA: Deep Canonical Correlation Analysis; DIABLO: Data Integration Analysis for Biomarker discovery using Latent cOmponents; JIVE: Joint and Individual Variation Explained; MKL: Multiple Kernel Learning; MOFA: multi‐omics factor analysis; NMF: Nonnegative Matrix Factorization; O2PLS: Two‐way Orthogonal Partial Least Squares; PLS: Partial Least Squares; VAE: Variational Autoencoder; WGCNA: Weighted correlation network analysis.

**FIGURE 3:**
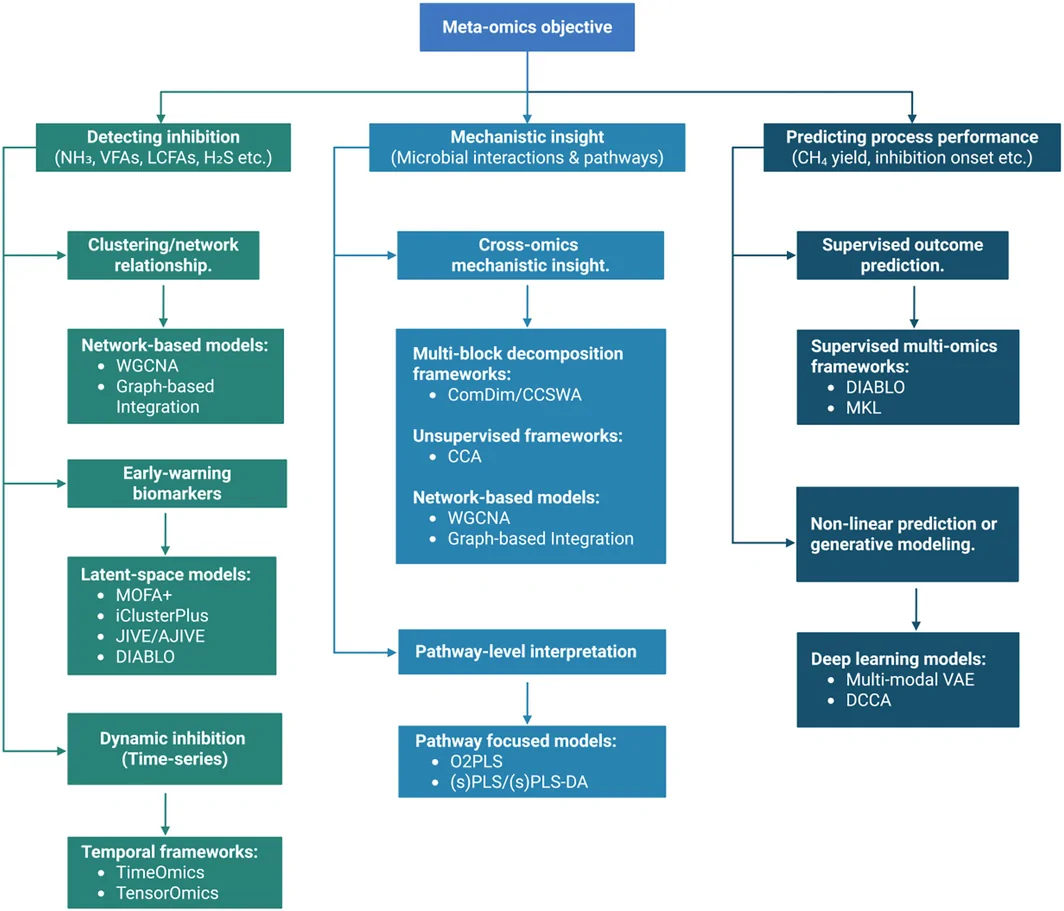
Flowchart for selecting suitable multi‐omics integration framework. AJIVE: Angle‐Based Joint and Individual Variation; CCA: Canonical Correlation Analysis; CCSWA: Common Components and Specific Weights Analysis; ComDim: Common Dimensions; DCCA: Deep Canonical Correlation Analysis; DIABLO: Data Integration Analysis for Biomarker discovery using Latent cOmponents; JIVE: Joint and Individual Variation Explained; MKL: Multiple Kernel Learning; MOFA: Multi‐omics factor analysis; NMF: Nonnegative Matrix Factorization; O2PLS: Two‐way Orthogonal Partial Least Squares; PLS: Partial Least Squares; VAE: Variational Autoencoder; WGCNA: Weighted correlation network analysis. Created in BioRender: https://BioRender.com/ooivgyg.

A flowchart for selecting a suitable multi‐omics integration framework based on study objectives is presented in Figure 3. While frameworks like PLS/O2PLS, CCA, NMF, WGCNA, graph‐based integration and tensor decomposition have already been widely applied to AD (Cardona et al. 2020; Lü et al. 2022), suitable methods like DIABLO, MOFA+, ComDim/CCSWA, TimeOmics, TensorOmics, multi‐modal VAE, DCCA, MKL, iClusterPlus and JIVE/AJIVE have not yet been widely applied.

#### 3.2.2 Integration Approaches

**FIGURE 4:**
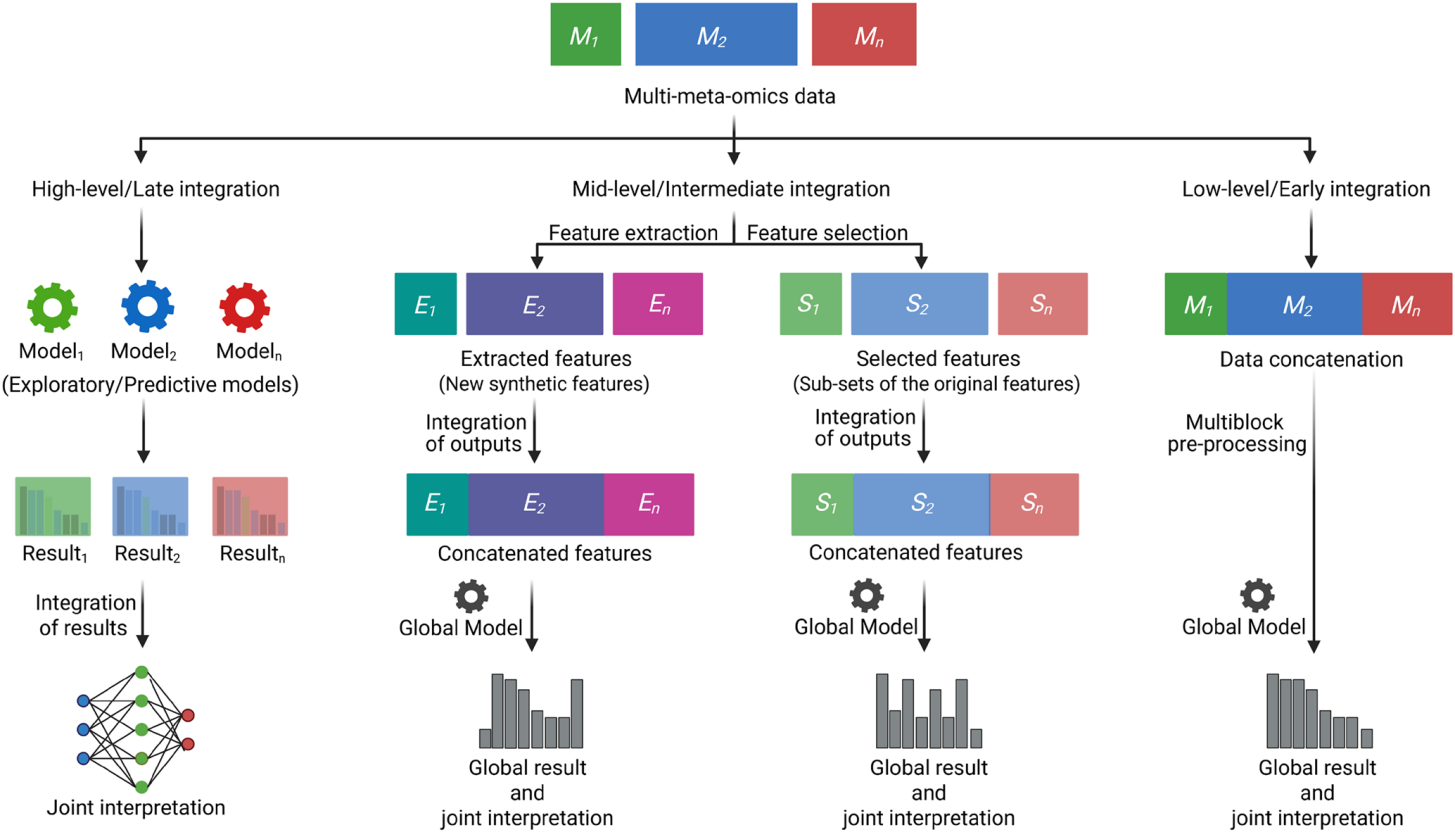
Schematic illustration of the three approaches for multi‐meta‐omics data integration. High‐level/late integration involves the combination of the results obtained from each meta‐omics model. Mid‐level/intermediate integration involves an initial dimensionality reduction (feature extraction or feature selection) before data fusion. In low‐level/early integration, meta‐omics data blocks are firstly concatenated into a multiblock and then processed and analysed together. These strategies are applied to gain a comprehensive understanding of the complex AD bioprocess. Created in BioRender: https://BioRender.com/f1cc8pn.

Generally, there are three approaches of integrating meta‐omics data (Figure 4). These approaches include high‐level/late integration, mid‐level/intermediate integration and low‐level/early integration (Jendoubi and Engel 2021; Azcarate et al. 2021; Brown 2020). High‐level integration involves uniquely the combination of the results derived from each meta‐omics data block models. This implies that independent models of each data block are established before integrating only the obtained results for a joint interpretation using visualization tools. As an example, fitting results from meta‐omics analyses unto a reference network map such as biological networks (metabolic networks, genetic network, protein interactions) enables a multi‐omics integration, visualization and interpretation (Jiao et al. 2026; Puig‐Castellví et al. 2022). In the reference network representation such as the Kyoto Encyclopaedia of Genes and Genomes (KEGG) network (Kanehisa et al. 2019) nodes often represent genes, proteins or metabolites and the edges signify the functional links between the nodes. Visualization of the result can aid interpretability of the biological processes or indicate regulatory associations. High‐level integration is a more flexible approach as samples (and the sample size) can be different between blocks. However, this method suffers from the obvious loss of underlying interactions between the different meta‐omics because the models are done separately before integration (Smilde et al. 2022). Furthermore, high‐level integration can be classified as knowledge‐based because it is highly dependent on external databases, reference networks or prior biological knowledge for integration, annotation and interpretation. Recent developments in computational statistics and chemometrics are rapidly shifting meta‐omics integration towards data‐driven or model‐based approaches.

Data‐driven or model‐based multi‐omics integration uses statistical models and machine learning to detect patterns, trends and establish links between and within multi‐omics data with or without prior biological knowledge (Noor et al. 2019). Both mid‐level and low‐level integration are data‐driven or model‐based integration. Mid‐level or intermediate integration involves an initial features extraction and/or selection from each meta‐omics dataset to lessen noise contribution from each block, decrease data dimensionality and complexity. Thereafter, the extracted/selected features are concatenated and fitted into a global model for an inclusive interpretation. Mid‐level integration combines both knowledge‐based and data‐based integration approaches. Dimension reduction can sometimes be influenced and driven by prior biological knowledge. However, deciding on the number of components to keep or how many variables to keep during variable extraction or selection is demanding. MOFA+ (Argelaguet et al. 2018) and NMF (Lin and Boutros 2020) are some methods for mid‐level integration. Like the high‐level integration, this approach can give lesser or no insight into the important links between the initial meta‐omics data blocks. As such, biological interpretation can be very tedious and/or inconclusive because the selected or combined features may not be related.

Low‐level integration is an approach where meta‐omics data blocks are firstly concatenated into a multiblock and then processed and analysed together. This method preserves the underlying interactions between the different meta‐omics. Unlike the high‐level integration, this approach considers that the same samples are in the different blocks. This is important because low‐level approach assesses the relationship between the variables and the data blocks. However, this approach increases dimensionality and might not account for the varied distribution across the blocks that can cause severe bias. Consequently, multiblock pre‐processing is very important after the datasets are combined (Campos and Reis 2020). Scaling (block variance scaling, block rank scaling) is commonly applied on the multiblock to avoid any block dominating while the variance ratio between intra‐block variables is conserved (Mishra et al. 2021). Its simplicity and ease of application make low‐level integration a common data integration approach. Additionally, this approach can reveal interactions between variables in a single block or across data blocks through multiblock models. Interestingly, dimensionality reduction can be applied across the multiblock after integration and block scaling to isolate relevant features or latent variables (Smilde et al. 2022). The advantage of this approach over mid‐level integration is that all the meta‐omics data blocks are considered at once. ComDim/CCSWA is a suitable framework for low‐level meta‐omics integration (Cariou et al. 2019; El Ghaziri et al. 2016).

### 3.3 Application of Integrated Meta‐Omics Analyses in Biomarker Discovery

**FIGURE 5:**
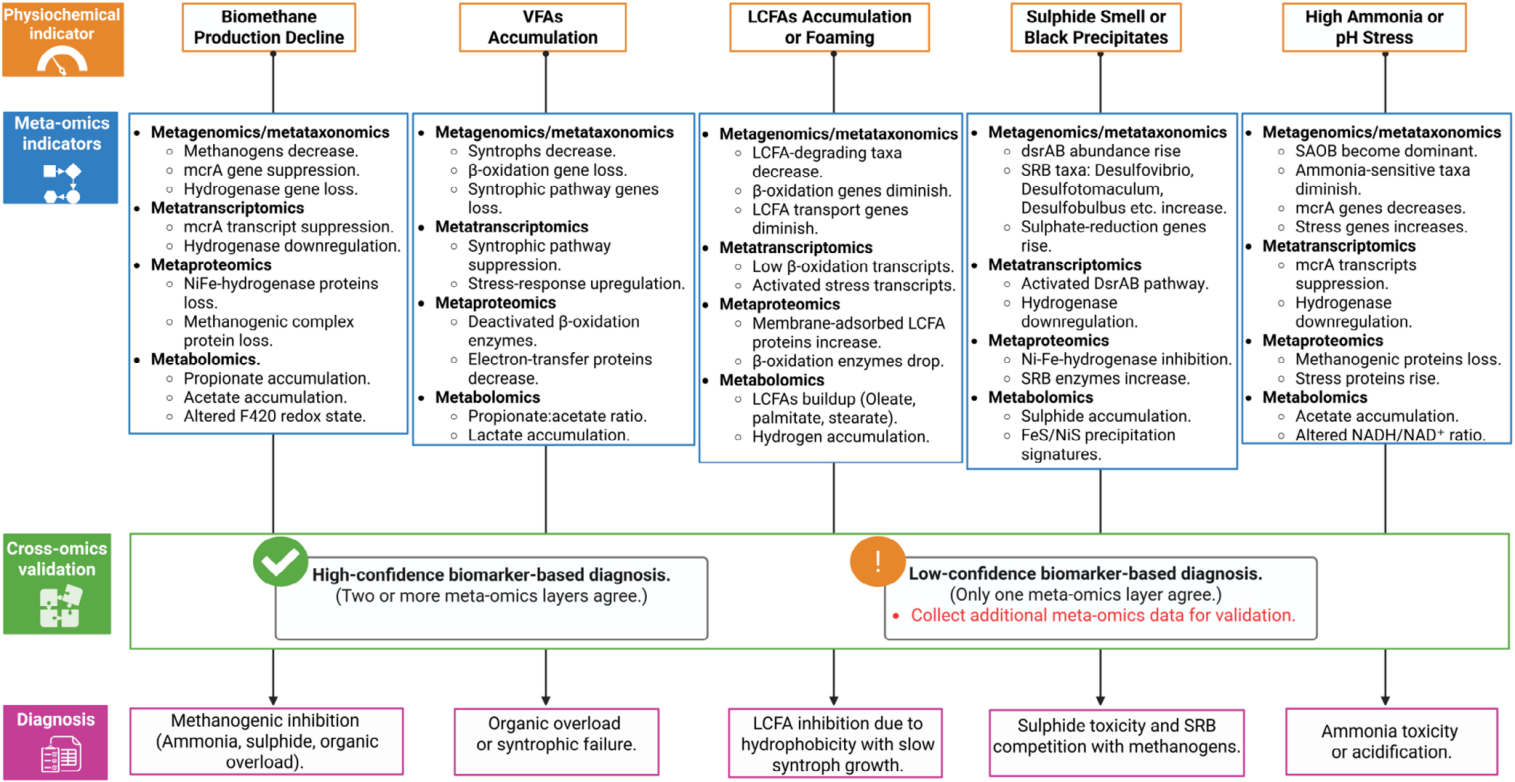
Biomarker‐based diagnostics with cross‐omics validation for anaerobic digestion. LCFAs: long chain fatty acids; SAOB: syntrophic acetate‐oxidising bacteria; SRB: sulphate‐reducing bacteria; VFAs: volatile fatty acids. Created in BioRender: https://BioRender.com/k2lqkxl.

Integrated meta‐omics biomarker discovery in AD is still emerging, especially when compared with clinical and medical research, where integrated approaches are already deeply embedded to identify robust biomarkers for diagnostics, precision medicine and disease stratification (Amini and Wang 2026). AD research, however, often relies on single meta‐omics biomarkers which are reproducible enough to be useful, but their predictive power and transferability across AD systems are limited. Integrated meta‐omics approach leads to discovery of more robust biomarkers by offering a multi‐omics perspective. A robust biomarker in AD should reliably reflects a specific inhibition mechanism or functional shift with coherent cross‐omics validation (Figure 5). Indeed, it must show strong specificity to a defined microbial guild or pathway like methanogenesis, syntrophic β‐oxidation, sulphate reduction, etc. and high sensitivity to operational stressors such as ammonia, sulphide, LCFA overload, organic or temperature shock. Crucially, a robust AD biomarker must be quantifiable with a clear mechanistic relevance, where a change must directly reflect a biochemical bottleneck or inhibition event. Finally, it must demonstrate a strong predictive value for early detection and diagnosis of inhibition or instability before performance decline (Lü et al. 2022; Poirier et al. 2020).

## 4 Conclusion

Meta‐omics remains an important tool to understanding, diagnosing and improving the AD bioprocess. However, no single meta‐omics approach is sufficient for a comprehensive analysis of the AD bioprocess. While this has led to the application of a multi‐meta‐omics approach that provides more valuable information and insight, focus is now on integrating the datasets or results generated from the various meta‐omics platforms for an all‐inclusive analysis and interpretation. Meta‐omics integration will advance our present understanding of the complex microbiome interactions in the AD bioprocess. While continued advancements in high‐throughput sequencing technologies and analytical platforms are anticipated, there remain challenges. One such challenge is managing the associated cost that arises from multi‐meta‐omics studies and downstream computational analysis. Another challenge involves dealing with the complexity and heterogeneity of meta‐omics datasets that often necessitate dimensionality reduction. Furthermore, with ease of interpretation being a key consideration in meta‐omics integration, easy‐to‐use computational and statistical tools and methodologies are needed to combine and analyse meta‐omics datasets, models and results. This will further accelerate the development of the AD bioprocess for sustainable energy generation and bioeconomy.

## Author Contributions

**Kevin Iyere Ehiosun:** conceptualization, writing – original draft, writing – review and editing, data curation, methodology. **Olivier Chapleur:** writing – review and editing, supervision, data curation, methodology. **Laurent Mazéas:** writing – review and editing, funding acquisition, data curation, supervision, methodology.

## Funding

This work was supported by Agence Nationale de la Recherche (ANR‐22‐CE43‐0014‐01).

## Conflicts of Interest

The authors declare no conflicts of interest.

## Data Availability

Data sharing not applicable to this article as no datasets were generated or analysed during the current study.
